# Formulation of a stable diesel microemulsion using eco-friendly ionic liquids and investigation of particle size and fuel properties as an alternative fuel

**DOI:** 10.1038/s41598-024-69856-9

**Published:** 2024-08-27

**Authors:** H. A. El Nagy, Mahmoud Abd El-Aziz Mohamed

**Affiliations:** 1 Chemistry Department, Faculty of Science, Suez Canal University, Ismailia, 41522 Egypt; 2 Abu Sultan Thermal Power Plant, East Delta Electricity Production Company, Ismailia, Egypt

**Keywords:** Ricinoleic acid, Ionic liquids (ILs), Microemulsion fuel, Synthesis, Diesel blends, Biofuel, Alternative fuel, Chemistry, Energy science and technology

## Abstract

Ecofriendly ionic liquids (ILs) were synthesized through amidation of ricinoleic acid, the main fatty acid in castor oil, followed by a quaternization reaction to solubilize ethanol in IL/diesel blends at different ratios. As a result, stable and highly renewable, low viscous microemulsion biofuels with high oxygen content were prepared. The prepared fuel samples combine the advantages of green ionic liquids and microemulsion properties. The chemical structures of ILs were confirmed with the aid of NMR and FTIR spectroscopy. DLS analysis revealed that the ethanol particles ranged in size from 8 to 18.1 nm in all samples. As ILs ratios decrease in microemulsion from 37 to 69%, the ethanol particle sizes increase from 10 to 25%. Ethanol shows good solubilization in diesel and IL-1 is more effective than IL-2 in ethanol solubilization at low percentages of ethanol due to more oxygen atoms besides three hydroxyl groups. The ternary phase diagram indicated that the microemulsion area in the case of using IL-1 is larger than that of IL-2. The fuel properties of the prepared microemulsions are nearly close to those of neat diesel and fall within the permitted range of ASTM D975. The viscosity and density values at low ratios of ILs are found to be very close to the values of the neat diesel at different temperatures. The prepared samples show a slight decrease in cetane number and heating value compared to diesel. However, they have improved flash points, cloud points, sulfur content, and acid value. The particle sizes were checked every week and the prepared samples showed high stability with the aid of the synthesized ILs. Moreover, the prepared microemulsions stayed in a transparent appearance for more than a year and no phase separation was observed.

## Introduction

When a diesel engine is used, harmful emissions such as carbon oxides (CO and CO_2_), nitrogen oxides (NO_x_), hydrocarbons (HC), sulfur oxides (SO_x_), and smoke are increased. These pollutants are the primary factors that cause an increase in greenhouse gases and harm to the ozone layer, driving research to find a brand-new renewable energy source to replace petroleum^1,2^.

The microemulsification technique is a method that can be utilized to minimize the issues related to the use of neat diesel fuels and convert them into acceptable biofuel^3–5^. The low cost of the product, simplicity of operation, and minimal requirement for fuel processing without chemical reactions are reasons why the microemulsification method is better than other methods. Another benefit of this approach is that it may be applied directly to diesel engines without requiring any engine system modifications^6^.

Typically containing both water and oil stabilized by surfactant and co-surfactant, microemulsions are translucent, low-viscous, thermodynamically stable solutions that form spontaneously^7^ and have droplet sizes less than 200 nm^8^. Microemulsions of colza oil, diesel, and water were found to be stable for more than nine months^9^. Testing of microemulsions as diesel fuel in the past has shown that they can reduce exhaust particulate matter and nitrogen oxide compound levels^10^.

According to numerous studies, adding alcohol to fossil fuels, such as methanol, ethanol, propanol, butanol, and pentanol, can lower particulate matter emissions. Alcohols are another type of renewable oxygenating fuel that contributes to the reduction of CO and soot emissions. They do, however, frequently split out and lessen the fuel's heating value^11,12^. Thus, the emulsification of alcohol in diesel and prevention of phase separation needs the use of surfactants or emulsifiers to enhance the blend stability^13^.

Ionic liquids (ILs) have gained popularity in recent decades as environmentally friendly solvents and surfactants in a wide range of applications^14,15^. One of the most significant uses for these substances is the use of ionic liquids in microemulsion systems. Depending on the cation and anion properties of ILs, They have been employed in microemulsions as polar or nonpolar phases and recently as an amphiphile^16^. Hydrophobic ILs have characteristics that are similar to the oil phase for the generation of microemulsions because ILs are safe for the environment and as a result have peculiar solvent properties^17^. Jan et al.^18^ studied the effect of different hydrophobic ionic liquid structures on the phase behavior of water/TX-100/IL microemulsions. Xu et al.^19^ used the 1-butyl-3-methylimidazolium hexafluorophosphate (BmimPF_6_) as a non-polar IL phase to study the BmimPF_6_/DMF/water system. The phase behavior of [C_4_mim][PF_6_] IL as a nonpolar phase in Tween 20/water microemulsion was examined by Seth et al.^20^ and Gao et al.^21^. Rai et al.^22^ demonstrated that water/IL microemulsions are formed when a zwitterionic surfactant is added to the ILs [C_4_mim][PF_6_] and [C_2_mim][NTf_2_] together with ethanol. The water/IL microemulsion is produced when ethanol is added because it increases the solubility of the surfactant and water in the IL. Additionally, studies have been conducted on the [C_4_mim][BF_4_] as polar IL in toluene microemulsion in the presence of Triton X-100, a nonionic surfactant by Gao et al.^23^ and Li et al.^24^. An example of IL microemulsions composed of 1-butyl-3-methylimidazolium tetrafluoroborate ([C_4_mim] [BF_4_], toluene, and ethanol made without the use of conventional surfactants was presented and characterized by Xu et al.^25^. Hydrophilic ionic liquids and hydrophobic ionic liquids with TX-100 (nonionic surfactant) and water^26^ were used to create microemulsions in a variety of organic solvents such as triethylamine^27^, toluene, xylene, and cyclohexane^24^.

Besides, the usage of ILs as surfactants is attracting researchers' attention more and more frequently. ILs with long alkyl chains act like good amphiphiles, making them perfect for the formation of specific microemulsions. Zech et al.^28^ prepared stable microemulsion using dodecane as the continuous phase, ethylammonium nitrate (EAN) as polar phase IL, and 1-hexadecyl-3-methyl imidazolium chloride, an IL with surfactant characteristics. The use of room-temperature ionic liquid (RTIL) with an imidazole head as a hydrophilic component and a lengthy hydrophobic tail for emulsion polymerization is discussed in the first paper in this field^29^. According to studies, the imidazolium cation has a long chain that serves as an emulsifying agent, enhances the solubility of ionic liquids in diverse media, facilitates the creation of emulsions, and has many forms and special features^30^. ILs based microemulsions for sludge treatment were successfully prepared, and the surface-active ILs [C_16_mim] Cl, which were synthesized by combining imidazole group and 1-chlorohexadecane, were utilised as a surfactant^31^. Using [C4mim][AOT] as surface active IL, benzene as a non-polar solvent, and various IL as polar solvents, Sarkar and colleagues effectively formulated an IL-oil microemulsion. Sarkar and colleagues synthesized [C4mim][AOT] by an anion exchange reaction between NaAOT and 1-butyl-3-methylimidazolium bromide [C4mim]Br^32^. Using IL as polar media, IPM as a non-polar solvent, and N,N-dimethylethanolammonium 1,4-bis(2-ethylhexyl) sulfosuccinate (DAAOT) as a surface active IL, Sarkar and coworkers have prepared a microemulsion that is stable at high temperatures^33^.

Ionic liquids have shown certain benefits over organic surfactants in a number of applications. Among them, melting points around 100 °C, high thermal and chemical stability, nonflammability, miscibility, and solubility with a wide range of solvents, low toxicity, non-corrosive, and recyclable qualities can be mentioned^34–36^. The ability to tailor the characteristics of ionic liquids as surfactants through careful selection of cationic and anionic components, as well as their capacity to form extremely stable micelles without the need for cosurfactants, is, however, their most significant characteristic^37^. Making ionic liquid-based microemulsions that combine the benefits of green ionic liquids and microemulsions is interesting^38,39^. Few researches used surface active ILs in diesel microemulsions. Given that diesel is a complex blend of hydrocarbons, it is critical to investigate the diesel microemulsion systems and their characteristics as a renewable fuel.

This work focuses on the effect of the synthesized ILs in the formation of diesel microemulsion. The synthesized eco-friendly ILs are prepared from ricinoleic acid which is the main fatty acid in castor oil. The synthesis of ILs uses castor oil as a starting ingredient, encourages sustainability, and lessens dependency on non-renewable resources. To emulsify ethanol in IL/diesel blends, the new ILs were used as emulsifiers. Dynamic light scattering (DLS) examinations confirm the stability of the microemulsion fuels and the phase diagram was also displayed. Furthermore, the impact of the quantity of synthesized ILs and cosurfactant on the solubilization of ethanol was examined. The fuel characteristics of diesel fuel and ASTM limit parameters were compared with the fuel characteristics of microemulsion fuel samples.

## Experimental

### Materials and instrumentation

Ethyl chloroformate, dicyclohexyl carbodiimide (DCC) and castor oil were acquired from Aldrich Chemical. All other reagents and anhydrous solvents were acquired from Sigma Merck with an analytical grade and utilized without additional purification.

The synthesized ionic liquids were identified using a nuclear magnetic resonance proton (^1^H NMR, Bruker Advance, DRX-400, 400 MHz, TMS) spectrophotometer and an infrared spectrophotometer (Bruker).

### Synthesis procedure of the castor oil based ionic liquids

#### Castor oil hydrolysis

KOH solution (196 mmol) in 40 mL of ethanol was added to castor oil (14 mmol), which was then refluxed for 4 h. The mixture was added to an ice cube, stirred, and then acidified with a sulfuric acid solution (20% v/v) till pH 2. By removing them from the aqueous layer using diethyl ether, ricinoleic acid was separated. Anhydrous sodium sulphate was used to dry the extract, and ricinoleic acid with an 80% yield was obtained by vacuum-evaporating the solvent.

#### Synthesis of 12-hydroxy-N, N-bis(2-hydroxyethyl) octadec-9-enamide (2)

The ricinoleic acid (1) (0.01 mol), diethanolamine (0.012 mol) and DCC (0.012 mol) as coupling agent and KOH as an alkaline catalyst (1% w/w) were mixed with ethanol at 0 °C with stirring well for 1 h. The mixture was left to stir at room temperature for an average of 12 h. A 30 ml solution of chloroform: methanol 1:1 was then added and the organic phase was separated, dried, and concentrated. The product (2), 12-hydroxy-N, N-bis(2-hydroxyethyl) octadec-9-enamide, was obtained as a brownish-yellow viscous liquid with a yield of 85%.

#### Synthesis of ionic liquid (IL-1)

Ethyl chloroformate (0.01 mol) was added after the product (2) (0.01 mol) had been dissolved in ethanol. The mixture was stirred over a 24-h reflux at 70 °C. The IL-1 product was then obtained by cooling the reaction to ambient temperature, removing the ethanol, washing it with ethyl acetate, filtering, and drying it. IL-1 was obtained as viscous light brown liquid yielding 89%.

#### Synthesis of ionic liquid (IL-2)

Benzyl bromide (0.01 mol) was added to a solution of product (2) (0.01 mol) in ethanol. At 70°C and with constant stirring, the mixture was refluxed for 24 h. After the reaction had reached room temperature, the ethanol was removed, followed by an ethyl acetate wash, filtering, and drying to produce the end product, IL-2 as viscous dark brown liquid with a yielding of 94%.

### Microemulsion samples preparation and the ternary phase diagram

In the presence of the synthesized ionic liquids (IL-1 and IL-2), which functioned as emulsifiers, diesel, and ethanol were combined to create the produced microemulsion fuels. The synthesized ionic liquids were dissolved in diesel on a weight basis, IL-1: diesel (26%, 13%, 0.87% & 0.65% labeled as m1(IL-1), m2(IL-1), m3(IL-1) & m4(IL-1), respectively. IL-2 was also mixed according to the previous ratios. The addition of ethanol (5%), diesel (77%) and cosurfactant (1-butanol) was according to weight ratios obtained from the previous work^40^ which was found to be the optimum ratio. These ingredients were combined at room temperature while being stirred at 850 rpm for ten minutes. The microemulsions were kept in airtight glass containers. The microemulsions were shown to be stable for more than one year without any phase separation.

A three-sided equilateral triangle with three vertices that each represent a portion of the three components of the microemulsion makes up the ternary phase diagram. The percentage of the diesel/ionic liquid (O + IL) was represented at the triangle's bottom vertex. In addition, the percentage of the ethanol phase (E) and the cosurfactant phase (B) were represented on the right and left vertices of the system's ternary phase diagram, respectively.

### Dynamic light scattering analyses

The size and distribution of the ethanol particles in the produced microemulsion samples were determined by photon correlation spectroscopy utilizing Dynamic Light Scattering (DLS) at 25 °C (particle size analyzer, Zetasizer device, Nano ZN, UK, Malvern P analytical Ltd) and at a fixed angle of 173°. Three different tests were performed on the prepared microemulsion samples.

### The physicochemical parameters

The properties of the prepared microemulsions were evaluated according to ASTM standards and compared with the properties of diesel and ASTM D975 standards. The kinematic viscosity and density have been measured using the Tamson Tv4000MkIL and Densitometer A. Kruss Optronic standards of ASTM D445 and D5002, respectively. The cetane index is calculated using ASTM D976. Abbe's refractometer was used to calculate the refractive index. After using distilled water to calibrate the refractometer, a small quantity of microemulsion was placed on it, and the refractive index was measured and recorded. The cloud point is tested using the ASTM D2500. The water content was measured using the ASTM D4006 standard, Dean Stark Stanhope-Seta. The heating values and flash points were determined in accordance with ASTM D4809 and ASTM D93 standards, Auto flash point Stanhope-seta 35,000–04, respectively.

## Results and discussion

**Scheme 1:**
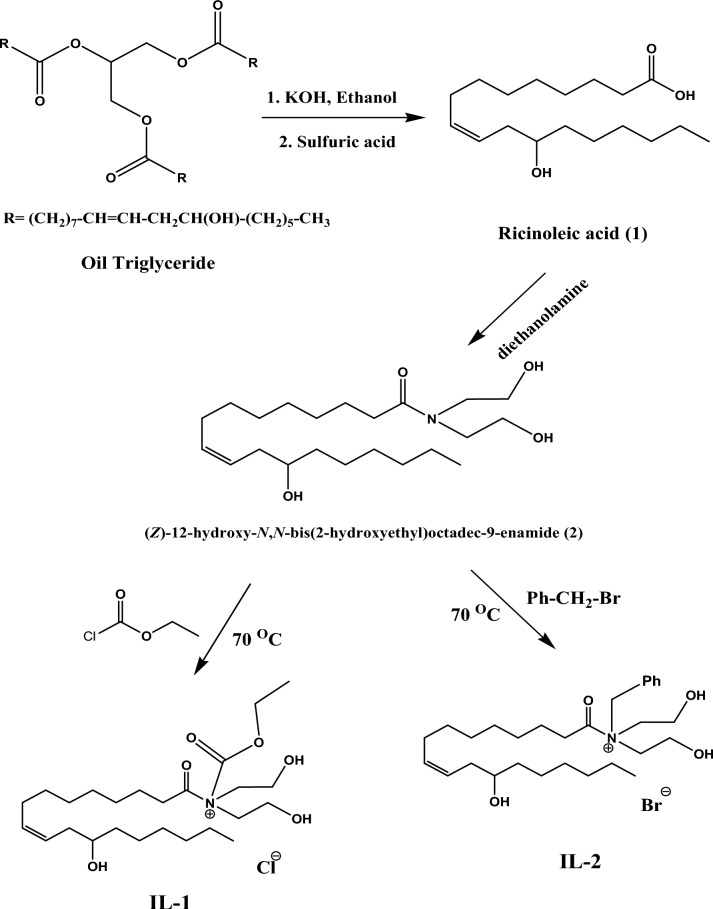
Synthesis of the ionic liquids (IL-1 & IL-2).

Ionic liquid-based microemulsions were prepared to be used as eco-friendly fuel. The new ionic liquids were synthesized depending on castor oil as a renewable material. Castor oil contains a considerable amount of ricinoleic acid, which was easily converted into ionic liquids through an amidation process and quaternization as indicated in Scheme 1. The ricinoleic acid composition comprises a long hydrocarbon chain and polar hydroxyl carboxyl groups, making it an amphiphilic compound. The new ionic liquids were employed as emulsifiers to disperse ethanol within the diesel fuel. As a result, the prepared fuel samples combine the advantages of green ionic liquids and microemulsion properties. Ionic liquids that come from organic materials often have minimal toxicity and acceptable compatibility with other solvents^41^.

During the formation of the microemulsion, emulsifiers assist in the expansion of the interface. Additionally, it can surround the ethanol particles that are broken down and dispersed in diesel, which stabilizes the dispersed phase in the continuous phase and increases the homogeneity of the microemulsion. Additionally, the emulsifier has an amphipathic effect that restricts and inhibits droplet agglomeration^42^.

### Spectroscopic characterization for the synthesized ionic liquids

^1^H-NMR, 400 MHZ, DMSO-d_6_ (ppm) of ricinoleic acid (1) presented at chemical shifts: 0.94–0.97 (t, 3H, CH_3_–(CH_2_)_4_–), 1.23 (t, 8H, CH_3_–(CH_2_)_4_–), 1.26 (t, 8H, CH==CH–CH_2_–(CH_2_)_4_–), 1.33 (q, 2H, CH_3_–(CH_2_)_4_– CH_2_), 1.96 (q, 2H, CH==CH–CH_2_–(CH_2_)_4_–), 2.01 (1H, OH), 2.26–2.29 (t, 2H, HO–CH–CH_2_–CH=CH), 3.29 (2H, CH_2_–CO), 3.41–3.42 (m, 1H, HO–CH–CH_2_–CH=CH), 5.35–5.39 (dd, 2H, CH==CH) and 11 (s, 1H, –CO–OH). FTIR (cm^−1^) shows a broad band at 3459 cm^−1^ (–OH, stretching). The peak at 3008 cm^−1^ corresponds to the =C–H stretching. The asymmetric and symmetric C–H (methylene and methyl groups) stretching are represented by the peaks at 2927 and 2857 cm^−1^, respectively. The bands at 1740 cm^−1^, 1457, 726 are attributed to (C=O acid, stretching), (–CH_2_–, bending), and (cis alkene), respectively.

^1^H-NMR, 400 MHZ, DMSO-d_6_ (ppm) of 12-hydroxy-N, N-bis(2-hydroxyethyl)octadec-9-enamide (2) presented at chemical shifts: 0.94–0.97 (t, 3H, CH_3_–(CH_2_)_4_–), 1.23 (t, 8H, CH_3_–(CH_2_)_4_–), 1.25 (t, 8H, CH==CH–CH_2_–(CH_2_)_4_–), 1.35 (q, 2H, CH_3_–(CH_2_)_4_–CH_2_), 1.96 (q, 2H, CH=CH–CH_2_–(CH_2_)_4_–), 2.00 (3H, 3OH), 2.26–2.28 (t, 2H, HO–CH–CH_2_–CH=CH), 3.31 (2H, CH_2_–CO–N), 3.40 (m, 1H, HO–CH–CH_2_–CH=CH), 3.43–3.46 (t, 4H, CH_2_–N–CH_2_), 4.01–4.04 (q, 4H, N–CH_2_–CH_2_–OH) and 5.33–5.37 (dd, 2H, CH=CH). FTIR (cm^−1^) shows a broad band at 3371 cm^−1^ (–OH, stretching). The asymmetric and symmetric C–H (methylene and methyl groups) stretching are represented by the peaks at 2926 and 2857 cm^−1^, respectively. The bands at 1620 cm^−1^, 1459, 1057, 719 are attributed to (C=O amide, stretching), (–CH_2_–, bending), (–C–N) and (cis alkene), respectively.

**Figure 1:**
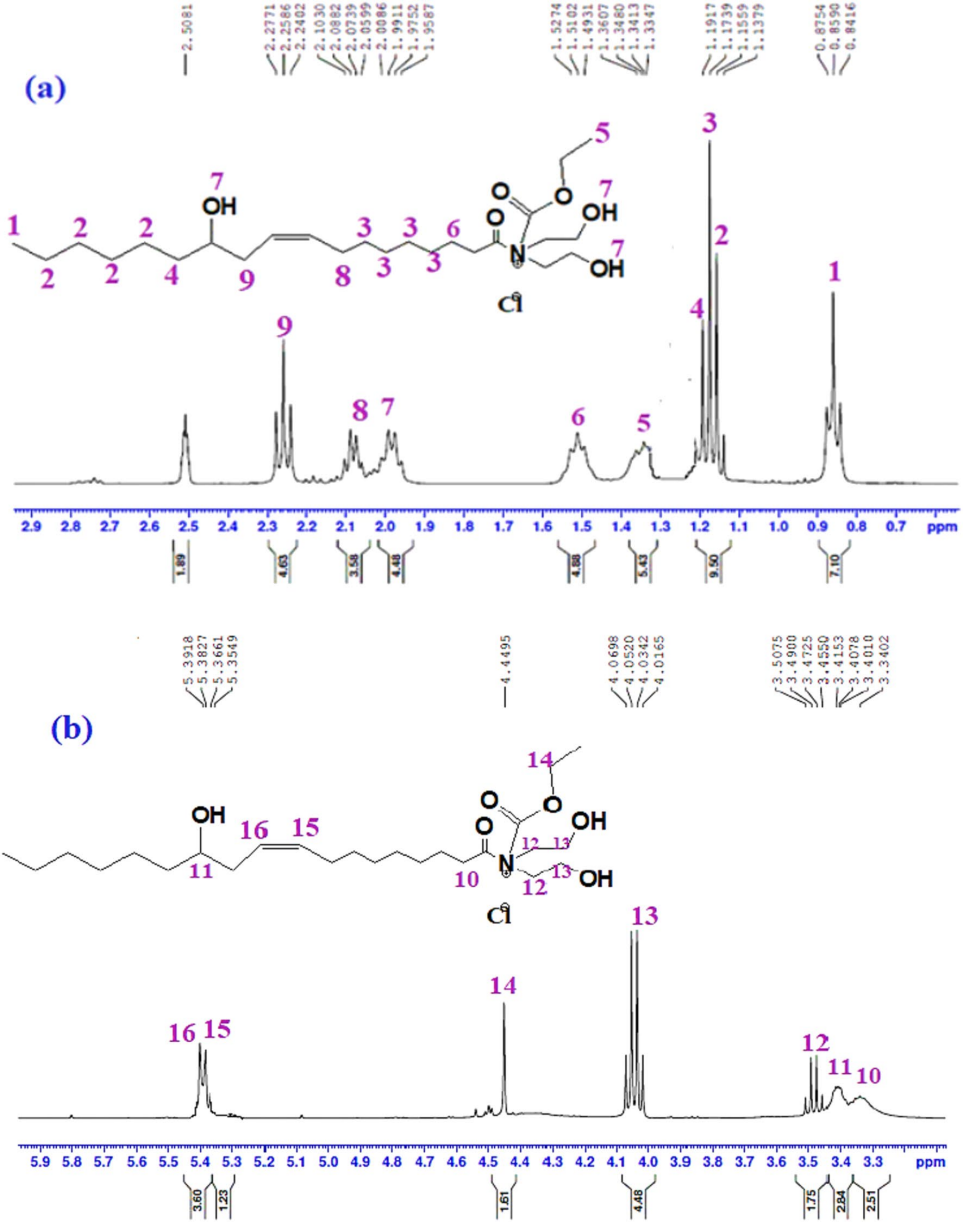
^1^H-NMR Chemical structure spectroscopic characterization of IL-1.

^1^H-NMR, 400 MHZ, DMSO-d_6_ (ppm) of IL-1 (Fig. 1a,b) presented at chemical shifts: 0.84–0.87 (t, 3H, CH_3_–(CH_2_)_4_–), 1.137 (t, 8H, CH_3_–(CH_2_)_4_–), 1.15 (t, 8H, CH==CH–CH_2_–(CH_2_)_4_–), 1.19 (q, 2H, CH_3_–(CH_2_)_4_– CH_2_), 1.33–1.34 (t, 3H, CH_3_– CH_2_–O–CO), 1.49–1.52 (m, 2H, CH_2_– CH_2_–CO–N^+^), 1.95 (3H, 3OH), 2.05–2.10 (q, 2H, CH==CH–CH_2_–(CH_2_)_4_–), 2.24–2.27 (t, 2H, HO–CH–CH_2_–CH==CH), 3.34 (2H, CH_2_– CH_2_–CO–N^+^), 3.40–3.41 (m, 1H, HO–CH–CH_2_–CH==CH), 3.45–3.49 (t, 4H, CH_2_-N^+^– CH_2_), 4.01–4.06 (q, 4H, ^+^N–CH_2_–CH_2_–OH), 4.44 (2H, CH_3_– CH_2_–O–CO) and 5.35–5.39 (dd, 2H, CH==CH). FTIR (cm^−1^) shows a broad band at 3351 cm^−1^ (–OH, stretching). The asymmetric and symmetric C–H (methylene and methyl groups) stretching are represented by the peaks at 2928 and 2857 cm^−1^, respectively. The bands at 1737 cm^−1^, 1660, 1446, 1067, 728 are attributed to (C=O ester, stretching), (C=O amide, stretching), (–CH_2_–, bending), (C–N stretching) and (cis alkene), respectively.

**Figure 2:**
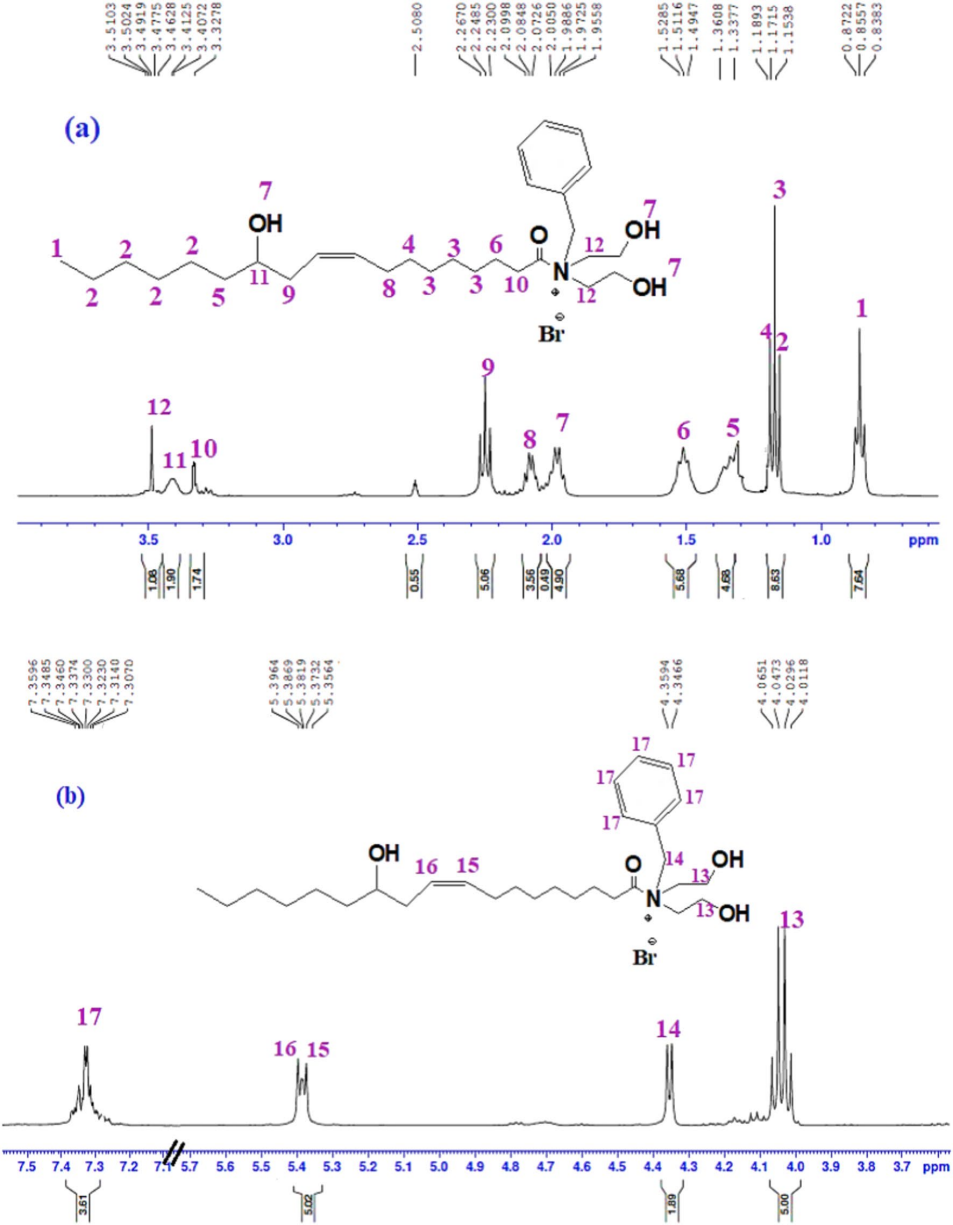
^1^H-NMR Chemical structure spectroscopic characterization of IL-2.

^1^H-NMR, 400 MHZ, DMSO-d_6_ (ppm) of IL-2 (Fig. 2a,b) presented at chemical shifts: 0.83–0.87 (t, 3H, CH_3_–(CH_2_)_4_–), 1.15 (t, 8H, CH_3_–(CH_2_)_4_–), 1.17 (t, 6H, CH==CH–CH_2_– CH_2_– (CH_2_)_3_–), 1.18 (2H, CH==CH–CH_2_–CH_2_–(CH_2_)_3_–), 1.33 (2H, CH_3_–(CH_2_)_4_– CH_2_), 1.49–1.52 (m, 2H, CH_2_– CH_2_–CO–N^+^), 1.95–1.98 (3H, 3OH), 2.07–2.09 (q, 2H, CH==CH–CH_2_–(CH_2_)_4_–), 2.23–2.26 (t, 2H, HO–CH–CH_2_–CH=CH), 3.32 (2H, CH_2_– CH_2_–CO–N^+^), 3.40–3.41 (m, 1H, HO–CH–CH_2_–CH==CH), 3.49–3.51 (t, 4H, CH_2_–N^+^– CH_2_), 4.01–4.06 (q, 4H, ^+^N–CH_2_–CH_2_–OH), 4.34–4.35 (2H, N^+^– CH_2_–Ph), 5.35–5.39 (dd, 2H, CH=CH) and 7.30–7.35 (Ar H). FTIR (cm^−1^) shows a broad band at 3324 cm^−1^ (–OH, stretching). The peak at 3010 cm^−1^ corresponds to the aromatic = C–H stretching. The asymmetric and symmetric C-H (methylene and methyl groups) stretching are represented by the peaks at 2926 and 2856 cm^−1^, respectively. The bands at 1663 cm^−1^, 1607, 1462, 1058, 721 are attributed to (C=O amide, stretching), (–C=C–, stretching), (–CH_2_–, bending), (C–N stretching) and (cis alkene), respectively.

### Ternary phase diagram

**Figure 3:**
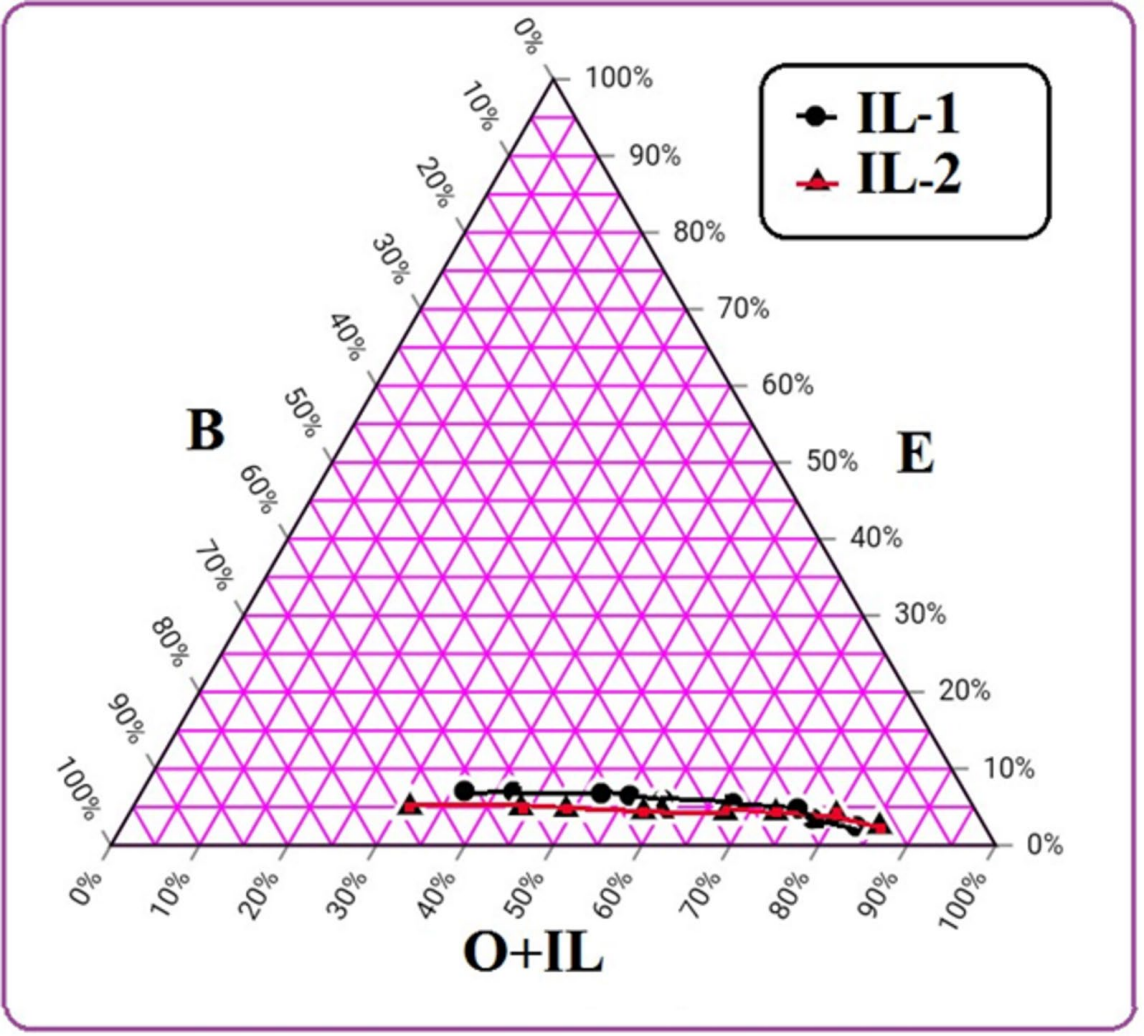
Ternary phase diagram of diesel/ionic liquid (O + IL), ethanol (E) and 1-butanol (B) at room temperature.

Figure 3 illustrates the phase diagram of room-temperature microemulsion systems made up of ethanol, 1-butanol as a co-surfactant, and diesel/synthesized ionic liquids (IL-1 & IL-2) (4:1). Different proportions of nonpolar and polar phases were mixed to determine the microemulsion area. The visual method used to identify the phase change is one of the most crucial aspects of microemulsion preparation. The dispersion of ethanol in diesel phase was studied at ratios 5% ± 2.5%. It was observed that the microemulsion area in case of using IL-1 as emulsifier is started from (2.5% E, 14.5% B and 83% O + IL-1) to (7.1% E, 56.5% B and 36.4% O + IL-1) while the microemulsion area in case of using IL-2 as emulsifier is started from (2.5% E, 12% B and 86.5% O + IL-2) to (5% E, 63.7% B and 31.3% O + IL-2). It was noticed from the ternary phase diagram that the microemulsion area in case of using IL-1 is larger than the microemulsion area in case of using IL-2. As the percentage of B increases in the microemulsion sample containing IL-1, the microemulsion area slightly increases while in case of IL-2, the microemulsion area is nearly constant.

### Ethanol solubilization in microemulsion

**Figure 4:**
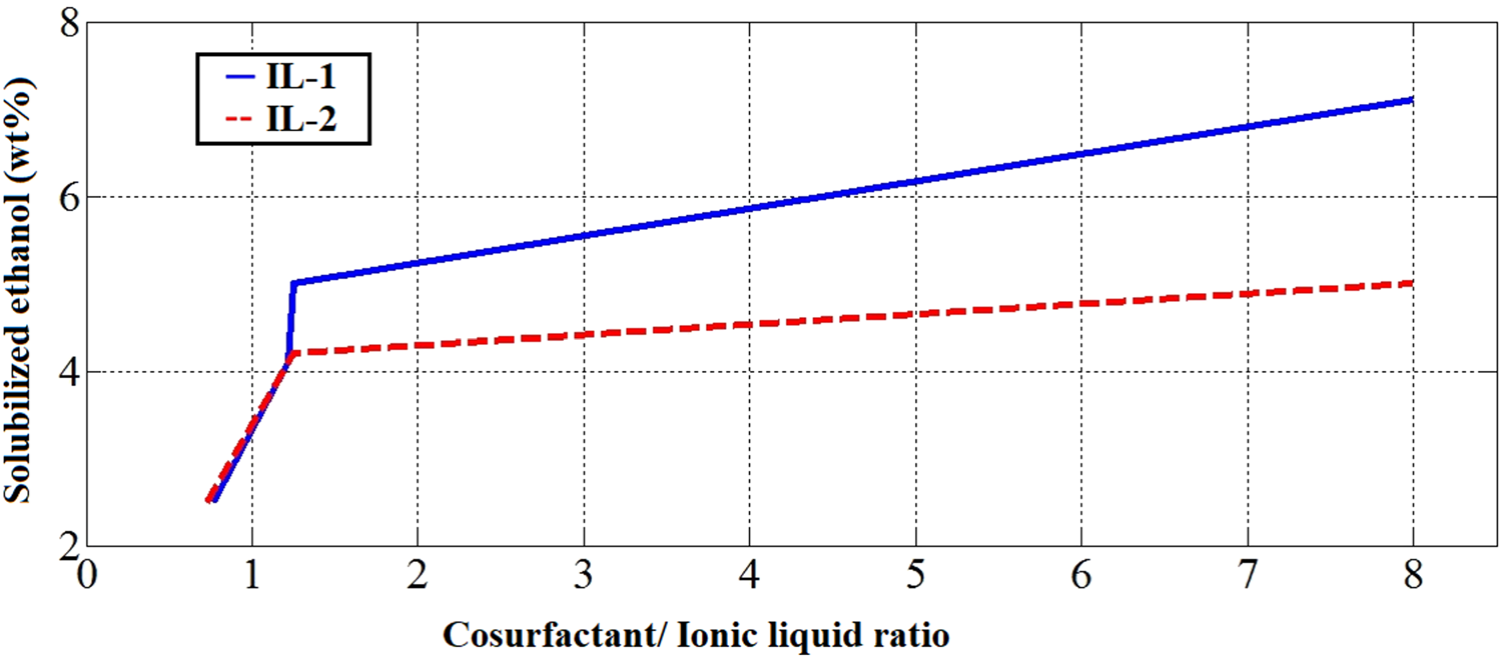
The Effect of synthesized ionic liquids and cosurfactant on ethanol solubilization.

As shown in Fig. 4, the amount of cosurfactant and ionic liquid is necessary for the efficient solubilization of ethanol in the microemulsion. The synthesized ionic liquids (IL-1 & IL-2) have the ability to make ethanol being solubilized within the diesel fuel with the aid of 1-butanol. But, their ability on ethanol solubilization varies. The amount of (IL-1 + cosurfactant) needed to solubilize 2.5% ethanol is 0.76 while the amount of (IL-2 + cosurfactant) needed is 0.73 to solubilize the same percentage of ethanol. The IL-2 and IL-1 nearly have the same effect on ethanol solubilization at low percentages of ethanol (2.5–4.1%). The two ionic liquids need the same amount of cosurfactant to solubilize 4.1% ethanol. Nevertheless, higher than 4.1% ethanol, the IL-1 is more efficient than the IL-2 in making ethanol solubilized within the diesel phase at the same ratios of cosurfactant/ionic liquids. Moreover, it was observed that there is a gradual increase in the amount of ethanol solubilized (> 5%) with the rise in the amount of (IL-1 + cosurfactant), in the case of IL-2, the amount of ethanol solubilized (> 5%) is slightly increases (nearly constant) and higher amounts from (IL-2 + cosurfactant). The hydroxyl groups in the synthesized ILs allow the formation of hydrogen bonds with alcohols in microemulsion and the polarity of ILs increases, as a result, enhancing the solubilization of ethanol in diesel. Therefore, the IL-1 is more preferred to solubilize ethanol because it contains three hydroxyl groups besides more oxygen atoms that increase the ethanol solubilization.

### The ethanol particle sizes

**Figure 5:**
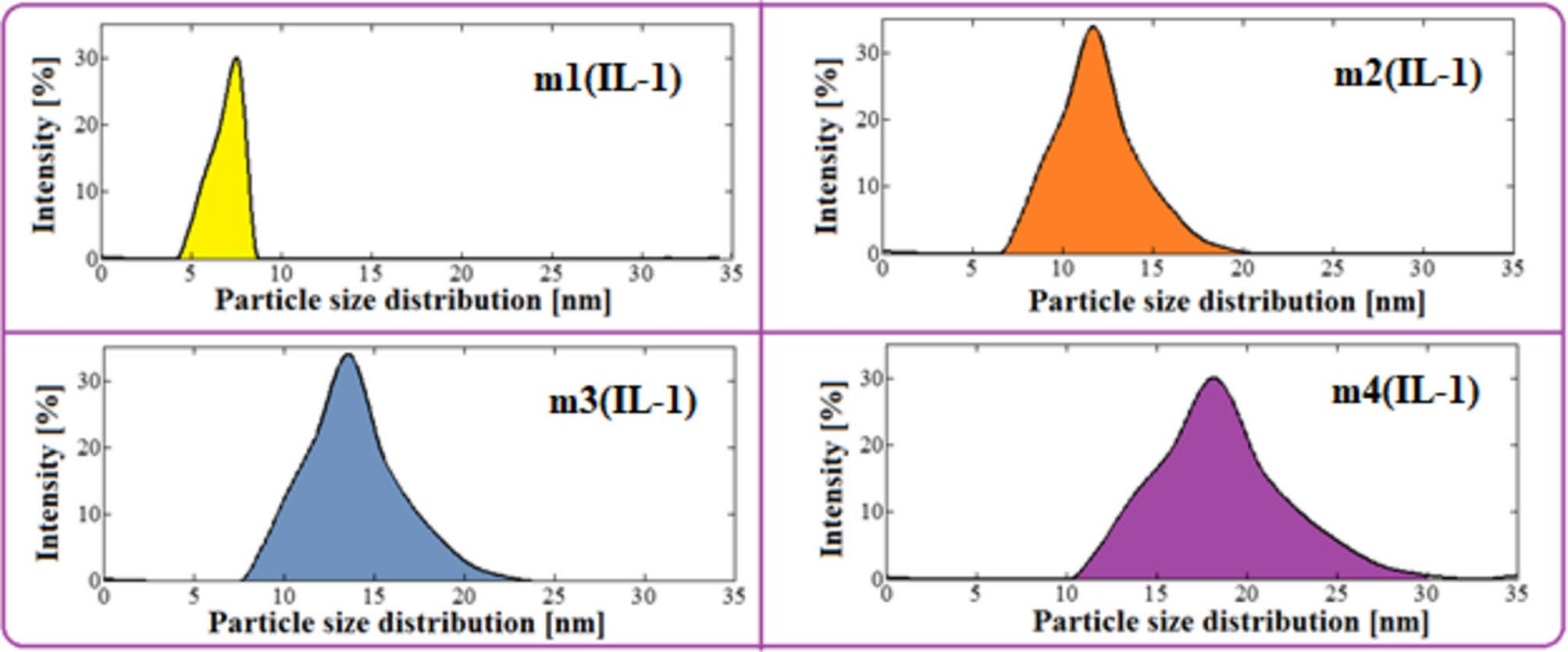
Particle size distribution of ethanol in fuel microemulsions using IL-1 as emulsifier.

**Figure 6:**
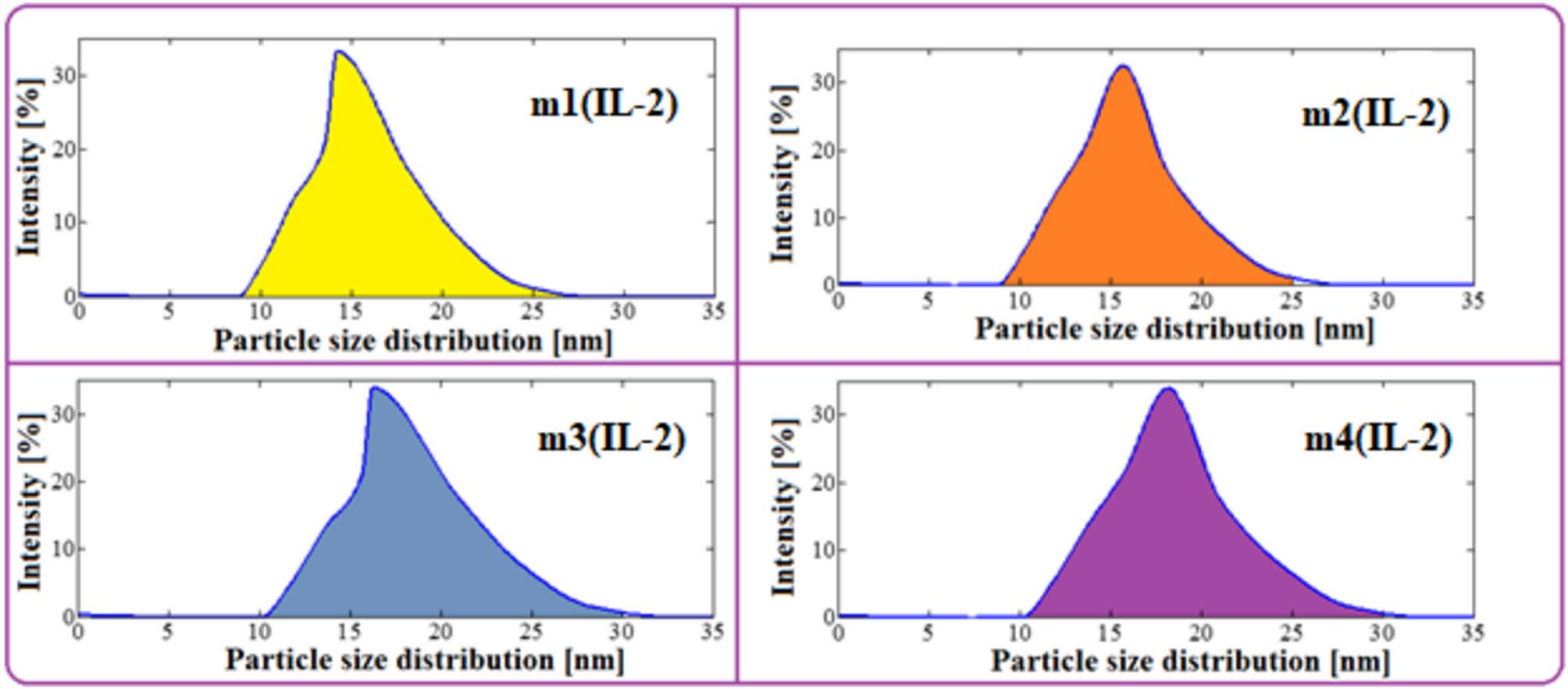
Particle size distribution of ethanol in fuel microemulsions using IL-2 as emulsifier.

Using the dynamic light scattering (DLS) technique, the size of the ethanol particles scattered in the fuel microemulsion as well as their size distribution were determined. The size and size distribution of the ethanol particles used in microemulsions with IL-1 and IL-2 as emulsifiers are shown in Figs. 5 and 6, respectively and in Table 1. The ethanol particles were found to be small, ranging in size from 8 to 18.1 nm in all samples which aid in the stability of the prepared microemulsions. This is due to the presence of hydroxyl groups in the synthesized ILs, increasing their polarity. Therefore, IL can adsorb on the interface and surround the ethanol particles tightly that are broken down and dispersed in diesel, as a result, enhancing the ethanol/diesel miscibility and increasing the homogeneity of the microemulsion^43^. Moreover, the hydroxyl groups in ILs allow the formation of hydrogen bonds with alcohols in microemulsion. In addition, It was observed that the particle sizes of ethanol in the case of using IL-1 as an emulsifier are lower than those in the case of IL-2 as an emulsifier at the same conditions. The microemulsion sample m1(IL-1) has the minimum particle size (8nm) which is approximately half the size of m1(IL-2) under the same conditions (IL/diesel 26%). As the ionic liquid ratios decrease in the microemulsion samples, the ethanol particle sizes increase, which is shown clearly in IL-1 samples. It was concluded that the IL-1 is more effective than the IL-2 in reducing the ethanol particle size. In addition, the ethanol particle size and its size distribution were close to each other in case of IL-1 and IL-2 at (IL/diesel 0.65%).

**Table 1:** The mean particle sizes for microemulsion samples.

Sample	IL-1				IL-2			
	m1(IL-1)	m2(IL-1)	m3(IL-1)	m4(IL-1)	m1(IL-2)	m2(IL-2)	m3(IL-2)	m4(IL-2)
Particle size (nm)	8	11.6	13.5	19	14.2	15.6	16.2	18.1
IL: diesel (wt%)	26	13	0.87	0.65	26	13	0.87	0.65

An increase in the percentage of diesel in the microemulsion samples means a decrease in the percentage of the ionic liquids (IL: diesel 26%, 13%, 0.87% & 0.65%). Table 2 shows the effect of Ionic liquids ratios on the ethanol particle size in microemulsion samples. It was observed that as the ratios of ionic liquids decrease in microemulsion (reduction ratio from 37 to 69%), the ethanol particle sizes increase (increasing ratio from 10 to 25% in case of IL-2 and from 48 to 150% in case of IL-1). Moreover, the effect of reducing the ratio of IL-2 on the particle size increases smoothly, while the effect of reducing the ratio of IL-1 has a clear effect on the increasing particle size.

**Table 2:** The effect of Ionic liquids ratios on the ethanol particle size in microemulsion samples.

IL-Reduction	(m1:m2)	(m1:m3)	(m1:m4)
	37.5%	58.5%	68.75%
Particle size increasing (IL-1)	45%	68.75%	137.5%
Particle size increasing (IL-2)	9.86%	14.08%	27.46%

### Stability of the microemulsion samples

Maintaining minimal engine modifications is one of the primary objectives of using fuel blends in engines, requiring blend stability and the development of a single-phase liquid system. Emulsifiers build up at the phase contact to lessen interfacial tension^44^. Emulsifiers develop up at the phase and interact to lessen interfacial tension. Emulsifiers work by forming a physical barrier or an attractive force between the dispersed phase and the emulsion droplets, thereby reducing the energy required to break the droplets apart and prevent them from collecting^45^.

**Figure 7:**
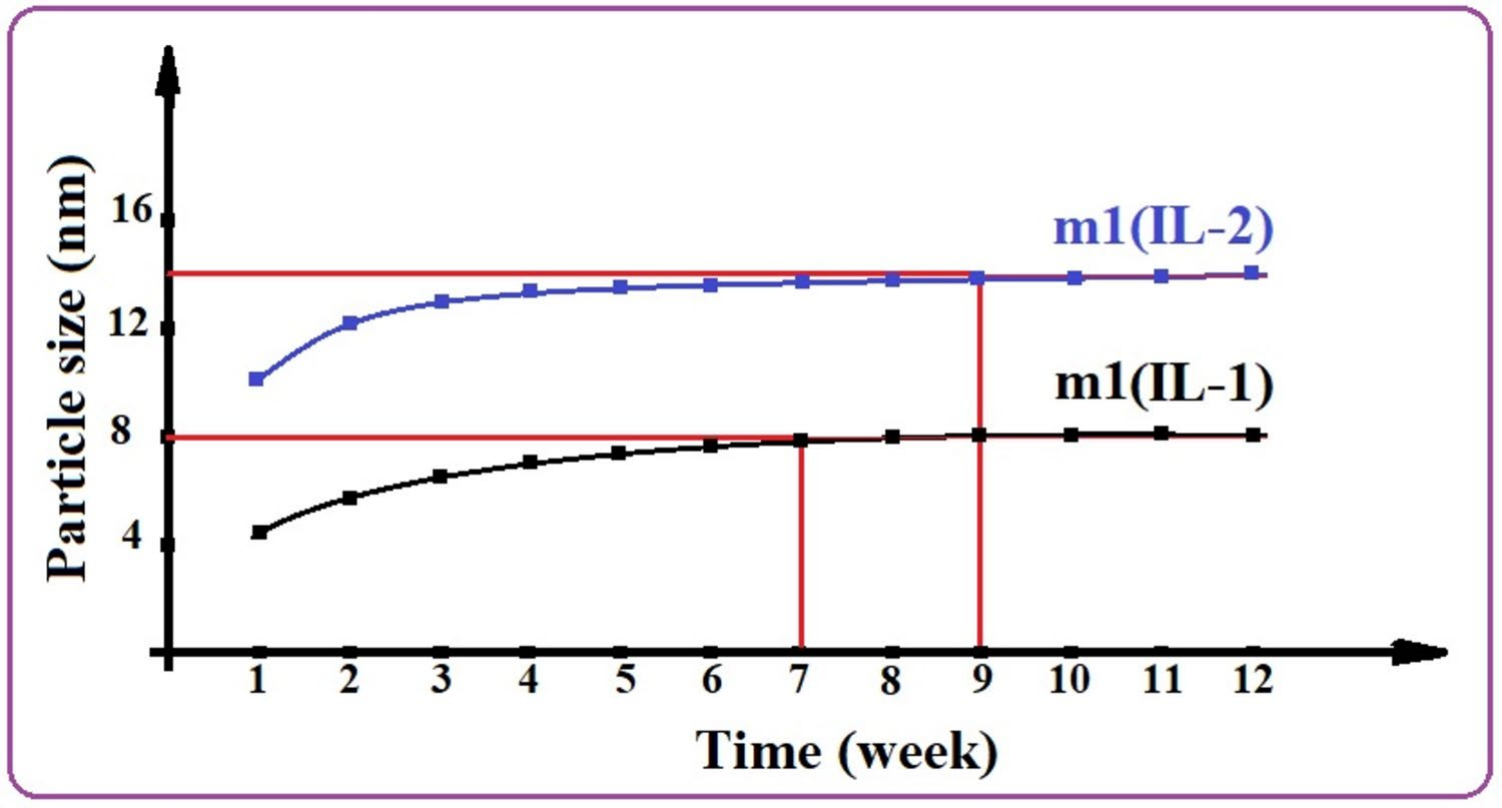
The stability of particle sizes for the prepared microemulsions with time.

The particle sizes of m1(IL-1) and m1(IL-2) samples were examined every week for three months. The microemulsion samples show significant stability as shown in Fig. 7. The particle size of the m1(IL-1) sample increases gradually until reaching the seventh week (8 nm). Its particle size was further measured after the seventh week and it was observed that it remained almost stable at 8 nm till reaching the twelfth week. The particle size of the m1(IL-2) sample reaches stability in the ninth week and remains stable at 14 nm till the twelfth week. Additionally, the samples were cooled up to − 10 °C and no phase separation was seen in the microemulsions, suggesting the development of stable microemulsion. Moreover, the prepared microemulsions stayed at our lab transparently for more than a year and no phase separation was observed. This proves the ability of the synthesized ionic liquids to enhance the microemulsion’s stability by keeping the ethanol particles dispersed in the diesel phase, as a result, enhancing the solubilization of ethanol.

### The physicochemical parameters of the prepared microemulsions

**Figure 8:**
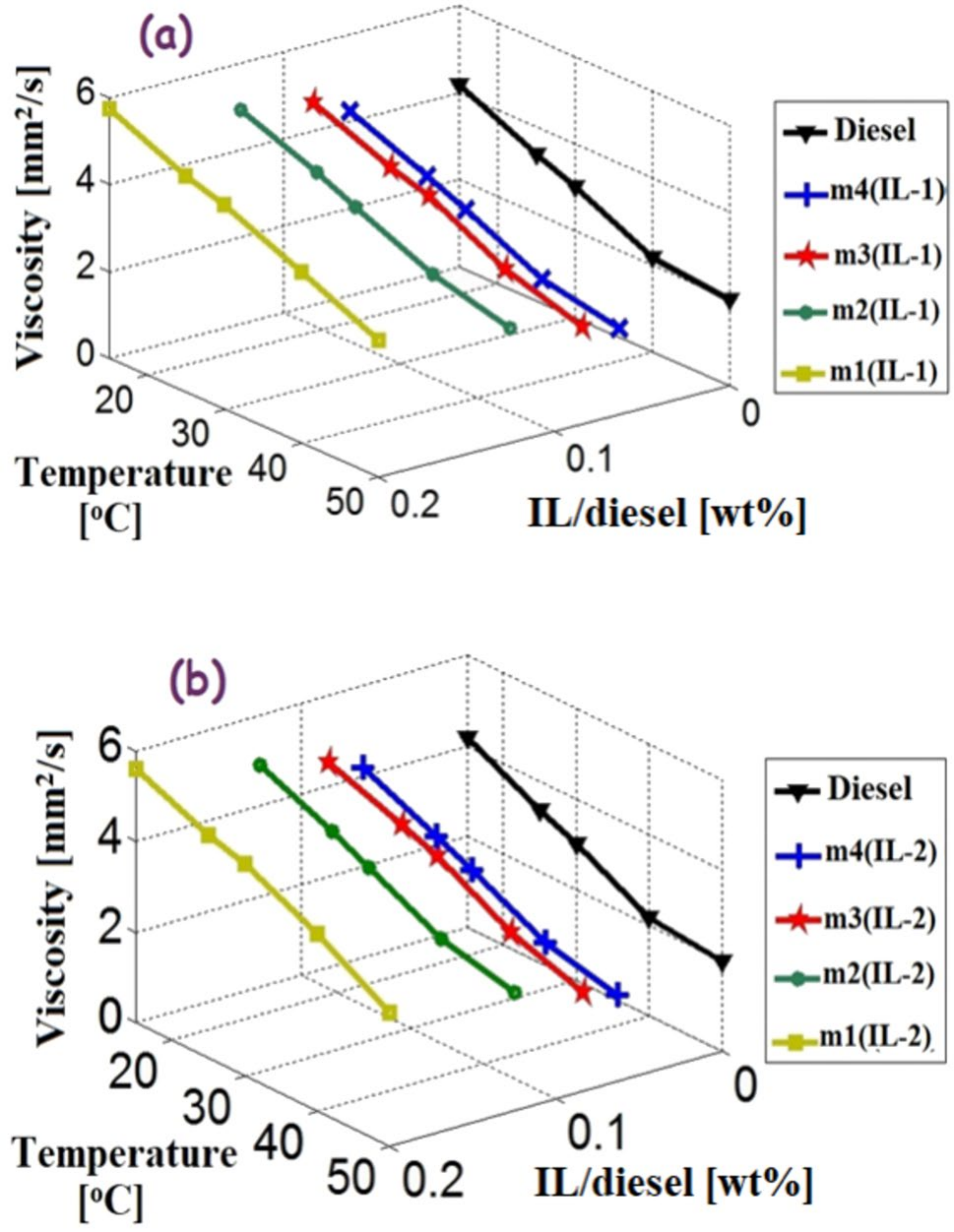
the relation among the viscosity of the microemulsion samples, the weight ratios of ionic liquids and the temperature (**a**) IL-1 samples and (**b**) IL-2 samples.

The fuel properties of the prepared microemulsions were measured and compared with the properties of the neat diesel and ASTM D975 standards. The viscosity of prepared samples was measured at different temperatures. Figure 8a,b shows the relation among the viscosity of the prepared samples, volume ratios of IL, and the temperature for IL1 and IL2 microemulsion samples, respectively.

As the temperature rises, the prepared samples become less viscous. Moreover, as the volume ratio of ionic liquid increases in microemulsion, the viscosity increases. For m1(IL-1) sample (contain 20% of IL1), at 40 °C, the viscosity increases by 3.96% compared to the viscosity of diesel. The viscosities of all IL1 and IL2 samples are within the ASTM D975 limits (1.9–4.1 mm^2^/s at 40 °C). In addition, the viscosities of m3(IL-1), m4(IL-1), m3(IL-2) and m4(IL-2) samples are found to be very close to the viscosity of the neat diesel at different temperatures. The IL1 and IL2 microemulsion samples nearly have the same effect but the samples of IL1 are slightly more viscous than the samples of IL2.

**Figure 9:**
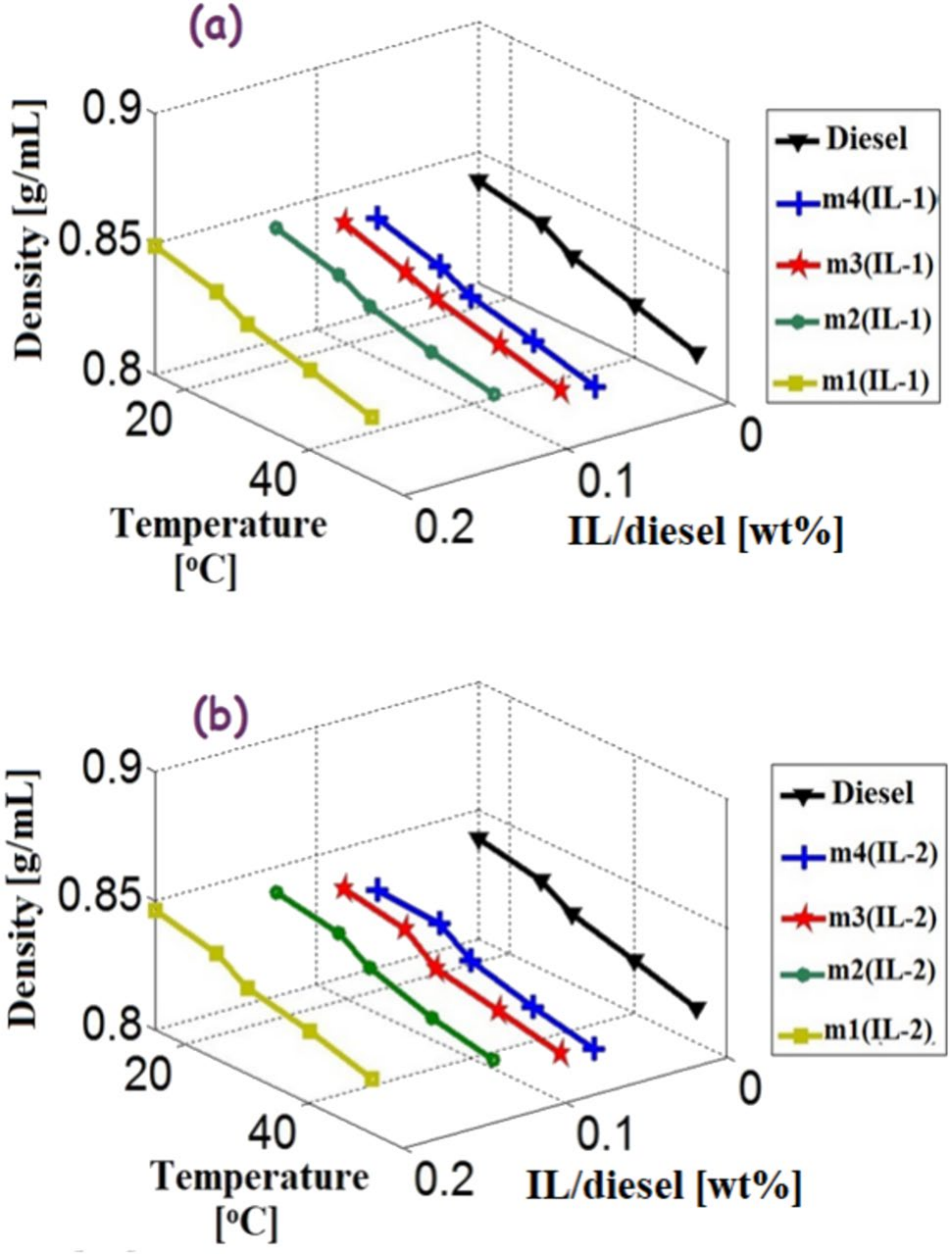
The relation among the density of the microemulsion samples, the weight ratios of ionic liquids and the temperature (**a**) IL-1 samples and (**b**) IL-2 samples.

The Fig. 9a,b show the relation among the density of the microemulsion samples, the volume ratios of IL and the temperature for IL1 and IL2 microemulsion samples, respectively. The densities of the prepared samples were found to decrease with the increase in temperature and decrease in the volume ratio of IL. As the ratio of IL increases by 20%, the density increases nearly by 1.2%. Also, the microemulsion samples were found to have densities that were close to those of neat diesel at different temperatures. The density and kinematic viscosity values of the fuel would affect the atomization, pumping of fuel into an engine, and eventually the evaporation of the fuel in an engine^46^. These attributes need to be close in value to the neat diesel as a result.

In the current study, the cetane number is replaced by the cetane index (ASTM D976). As indicated in Table 3, the prepared microemulsion fuels met the ASTM D975 acceptable minimum specifications and their cetane index was slightly lower than that of diesel because of the presence of alcohols in the microemulsion samples. The ricinoleic acid molecule also contains a double bond and free hydroxyl group, which lowers the cetane number. As the branching and unsaturation increase, the cetane number decreases^47^. However, the microemulsion fuels' lower cetane index suggested an ignition delay, they did not cause engine knock. Since alcohol has a lower heating value than diesel, the vapors that were produced had a lower peak temperature. In addition, the hydroxyl groups in the synthesized ionic liquids have higher ignitability, making it a possible option for use as emulsifiers and to improve ethanol/diesel microemulsion fuel.

**Table 3:** Fuel properties measurements for the prepared microemulsions.

Property	ASTM D975	Diesel	m1 (IL-1)	m1 (IL-2)	m4 (IL-1)	m4 (IL-2)
Cetane index	40	55	54	53.2	54.5	54.2
Heating value (MJ/kg)	–	45.6	41.17	41.3	42.6	42.8
Elemental analysis						
C%	–	85.5	76.44	76.72	78.79	79.2
H%		11.5	11.66	11.55	11.79	11.82
N%		–	0.43	0.38	0.12	0.11
S%		2	1.23	1.23	1.4	1.4
O%		–	8.48	7.26	6.37	6.01
Refractive Index	–	1.46	1.354	1.359	1.402	1.411
@ 25 °C						
Cloud point	–	− 3.3	− 10	− 10	− 9	− 9
(°C)						
Acid value (mg KOH/g)	–	–	0.434	0.454	0.304	0.323
Water and sediments (%v/v)	0.05	0.04	0.15	0.16	0.18	0.181
Flash point (°C)	52	56	61	60	59	57

One important property that is utilized to determine how much energy is produced when the fuel burns fully is its heating value. Results in Table 3 indicate that compared to diesel (45.6 MJ/kg), the heating values of the m1(IL-1), m1(IL-2), m4(IL-1) and m4(IL-2) microemulsion samples were slightly lower (41.17, 41.3 MJ/kg, 42.6 & 42.8, respectively). This is explained by the fact that these samples contained more oxygen, which naturally decreased their heating values. Moreover, the oxygen content (wt%) increased from 0% in pure diesel to 8.48% and 7.26% in microemulsion fuel after using IL-1 and IL-2, respectively, which enhance the complete combustion, as a result, reduce harmful emissions. Also, the sulfur ratio is decreased from 2 to 1.23%. Thus, microemulsion fuels are regarded as ecofriendly fuels.

An optical indicator of a substance’s ability to bend light beams is called its refractive index. The refractive index values of the microemulsions prove the transparency of the samples. Because ionic liquids and cosurfactant are present, microemulsions appear transparent or translucent yellow in colour. This illustrates how the microemulsion has very little scattering and low refractive index and is nearly transparent in the visible spectrum.

The minimum temperature at which wax starts to crystallize in fuels and gives them a hazy appearance is known as the cloud point. Since solidified waxes can clog engine fuel filters and injectors, the fuel's cloud point needs to be low for proper operation^48^. The lowest temperature restrictions for cloud point are not specified by ASTM D975, however according to IS 1460, they should be 3 °C for winter fuels and 15 °C for summer fuels. The microemulsion samples showed lower cloud points compared to diesel fuel due to the unsaturated fatty chain in the synthesized ionic liquids which has lower melting points than the saturated fatty chain indicated working without cold starts problems^49^. In addition, introducing low-cloud-point chemicals, such as 1-butanol and ethanol, improved the system's cold flow characteristics.

The amount of free fatty acids in the fuel sample is indicated by the acid value. The engine's fuel supply system corrodes when the acidity number is high^50^. The acid values for the prepared samples were found to be within the permitted level as indicated in Table 3. Both EN14214 and ASTM-D6751 standard fuels have an acid value of 0.50 mg KOH/g or less.

The used castor oil has acid value of 2.8 mg KOH/g and the process of hydrolyzing it to extract ricinoleic acid helps in lowering the acid value to use it as good raw material for the synthesis of ionic liquids and the preparation of microemulsions.

the water content values of the prepared samples (0.15%, 0.16%, 0.18%, and 0.181% for m1(IL-1), m1(IL-2), m4(IL-1) and m4(IL-2), respectively) are higher than the water content of diesel fuel (0.04%). It follows that the microemulsion fuel's water content is primarily derived from the ethanol that makes up its composition.

Flash point is an important property for the storage and handling of combustible materials. It measures the degree of inflammability or the extent of catching fire easily from the fuel^51^. The prepared samples show higher flash points (61, 60, 59, 57 °C for m1(IL-1), m1(IL-2), m4(IL-1), and m4(IL-2), respectively) compared to diesel (56 °C) and ASTM requirements. The increase in flash points may be due to the addition of the synthesized ionic liquids which are derived from the castor oil (less volatile and has a high flash point of 230 °C) to the microemulsion samples. Consequently, the prepared microemulsions have improved stability with fire compared to the neat diesel, making them safer.

## Conclusions

Environmentally friendly and stable microemulsion fuels were prepared through solubilizing ethanol in IL/diesel blends. The synthesized ionic liquids were derived from ricinoleic acid as a renewable material to replace the toxic ionic liquids or emulsifiers synthesized from traditional organic materials. All samples had ethanol particles ranging in size from 8 to 18.1 nm, according to DLS analysis. The ethanol particle sizes rise from 10 to 25% as the ILs ratios in the microemulsion drop from 37 to 69%. Because the produced ILs have hydroxyl groups, they are more polar and encircle the dispersed ethanol particles in diesel, as a result, improving the miscibility of ethanol and diesel fuel and raising the microemulsion's homogeneity. Therefore, ethanol has good solubilization, and at low ethanol percentages, IL-1 is more effective than IL-2 in facilitating ethanol solubilization due to the presence of more oxygen atoms besides three hydroxyl groups. The weekly checks on the particle sizes revealed that the prepared samples, with the help of the synthesized ILs, exhibited remarkable stability. Additionally, there was no phase separation seen and the created microemulsions maintained their translucent look for almost a year. The microemulsion fuel characteristics are within ASTM D975 allowed range and are almost identical to those of neat diesel. When compared to diesel, the produced samples exhibit a slight drop in heating value and cetane number. They do, however, have better acid values, cloud points, sulfur contents, and flash points. It is revealed that the density and viscosity values at low IL ratios are extremely similar to the neat diesel values at various temperatures. The oxygen content (wt%) increased from 0% in pure diesel to 8.48% and 7.26% in microemulsion fuel using IL-1 and IL-2, respectively. Also, the sulfur ratio is decreased from 2 to 1.23%. Although the presence of an increasing amount of ionic liquid leads to a small particle size, it is preferable to use a small percentage of the ionic liquid for diesel with a slightly large particle size (19 nm) due to improving their fuel properties and to keep the Cl (Br) from increasing.

Because the prepared microemulsions enhanced the fuel properties and due to the highly renewable content, they were demonstrated to be a successful substitute for diesel. Furthermore, the preparation process is inexpensive and simple, and the mixing is done at room temperature. The research results offer a means to enhance the environmental impact of diesel fuel performance.

## Data Availability

The datasets used and/or analyzed during the current study are available from the corresponding author upon reasonable request.
